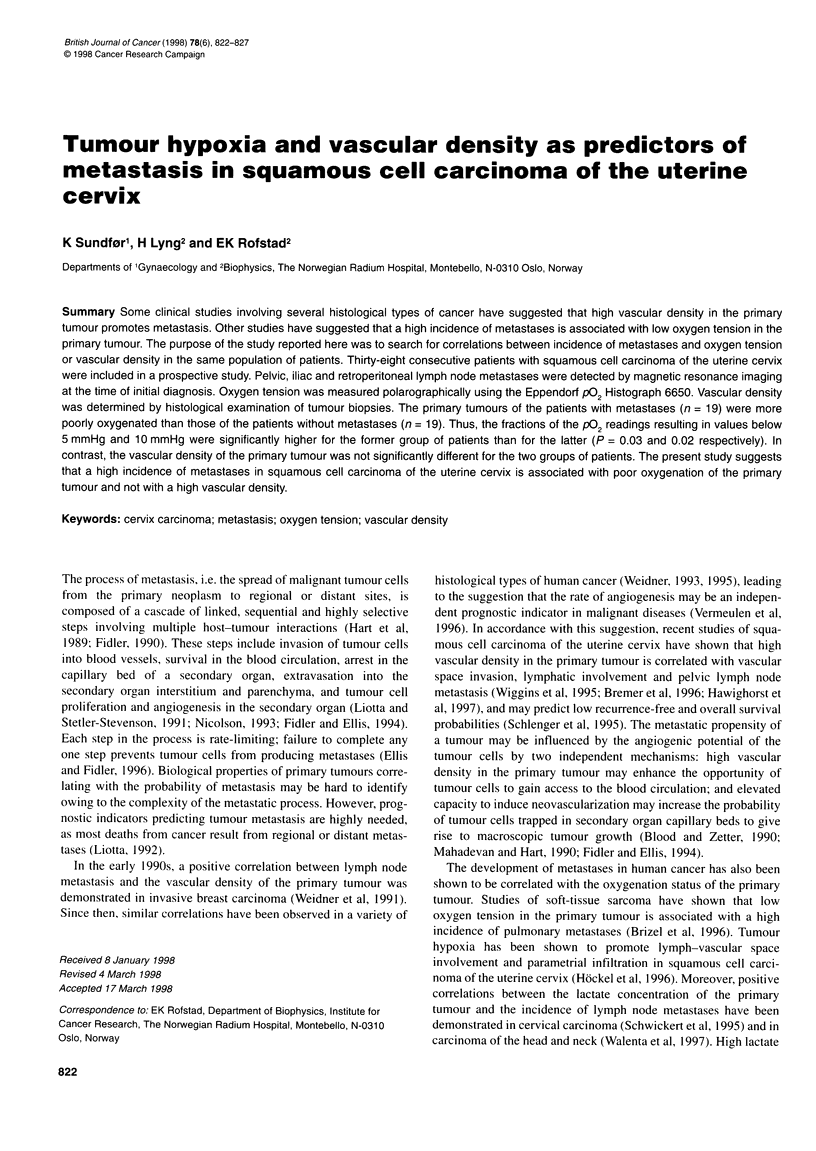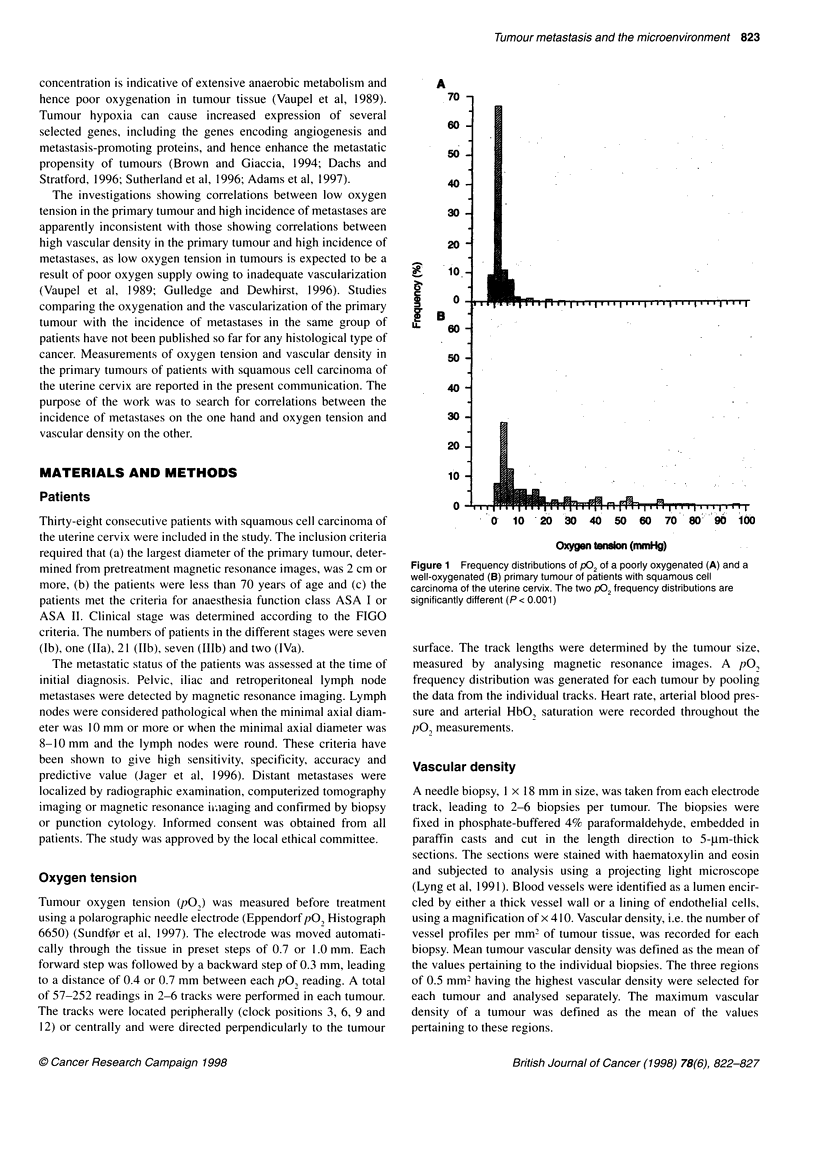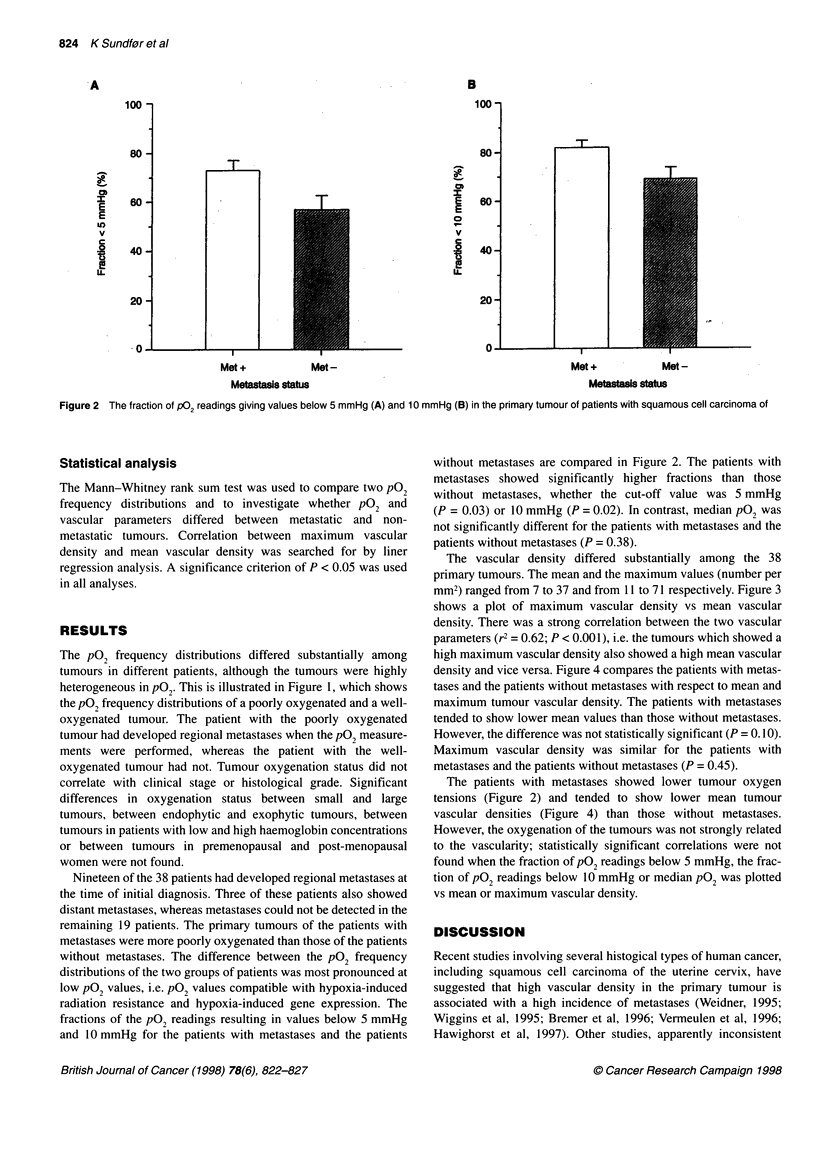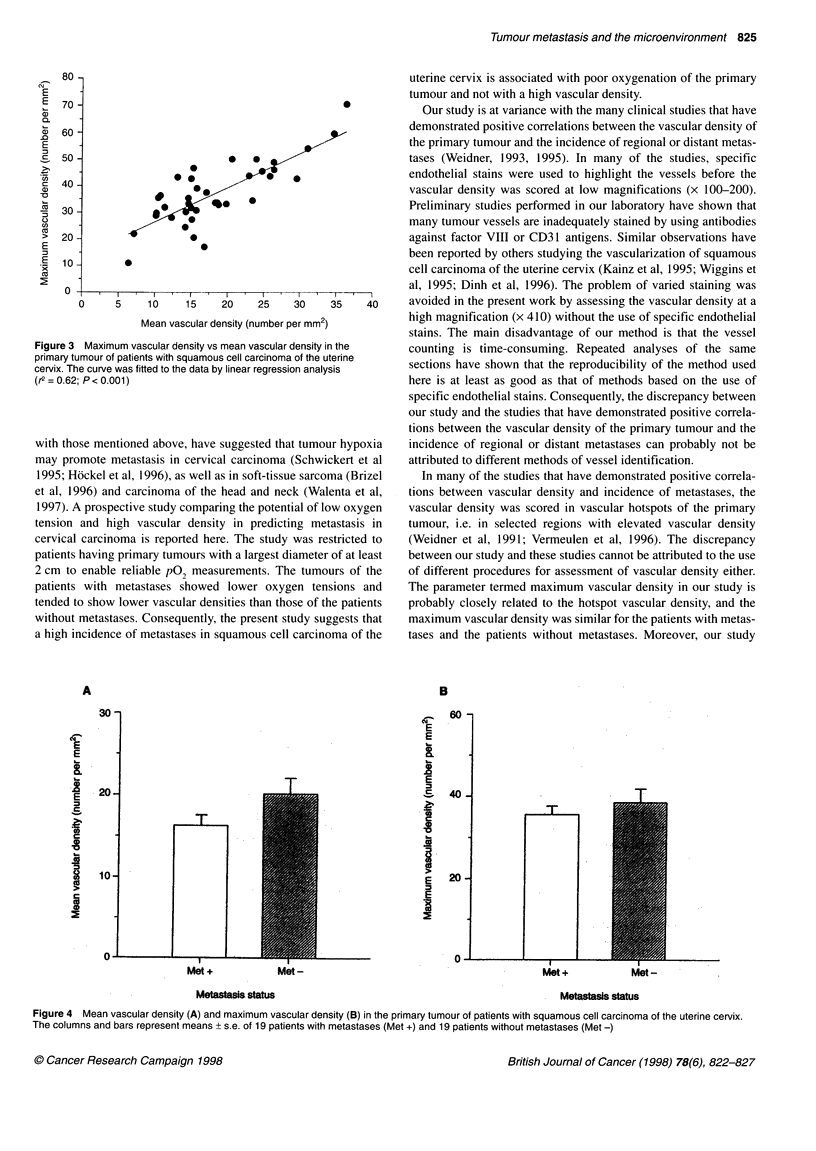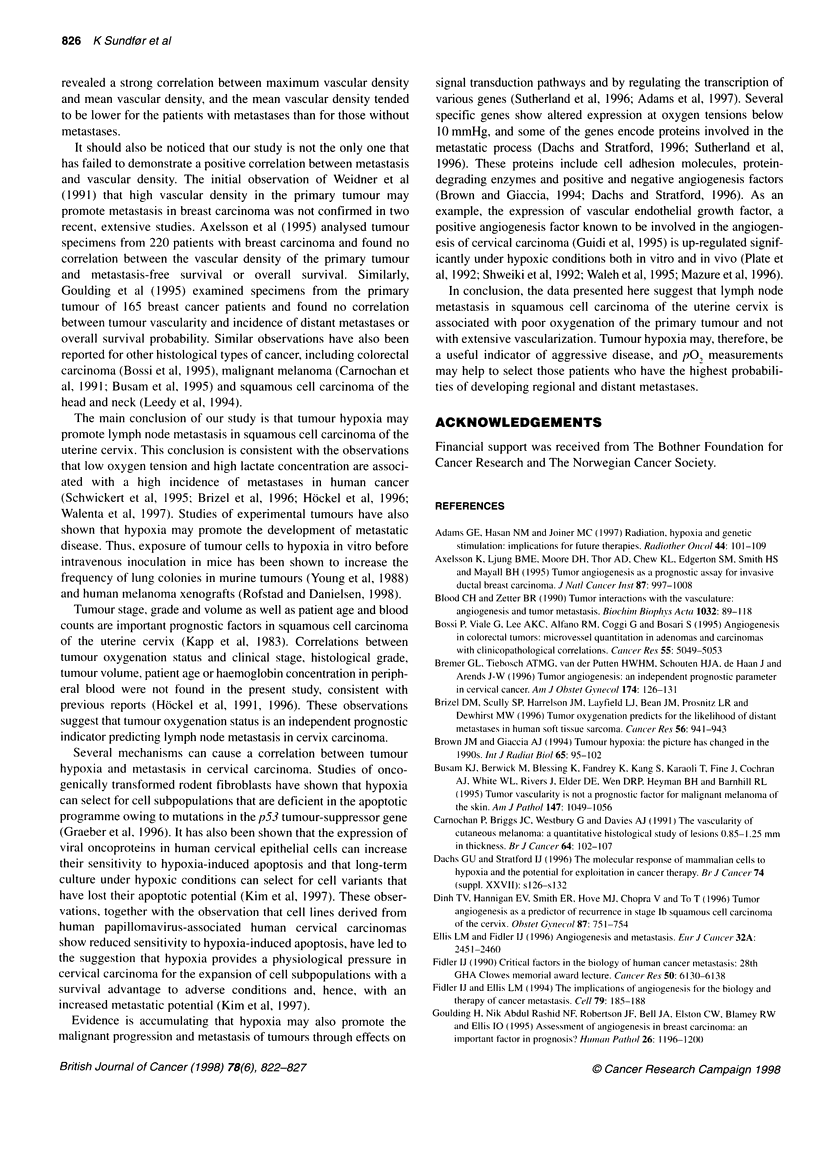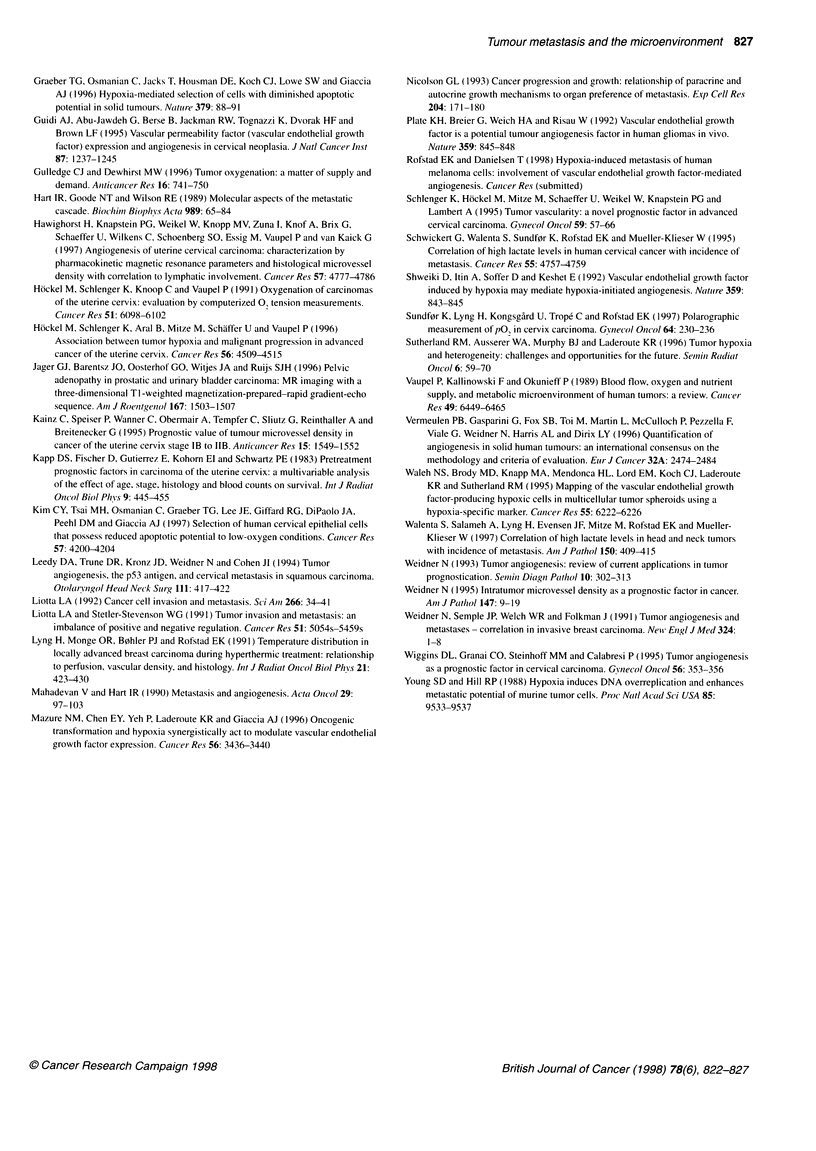# Tumour hypoxia and vascular density as predictors of metastasis in squamous cell carcinoma of the uterine cervix.

**DOI:** 10.1038/bjc.1998.586

**Published:** 1998-09

**Authors:** K. Sundfør, H. Lyng, E. K. Rofstad

**Affiliations:** Department of Gynaecology, The Norwegian Radium Hospital, Montebello, Oslo.

## Abstract

Some clinical studies involving several histological types of cancer have suggested that high vascular density in the primary tumour promotes metastasis. Other studies have suggested that a high incidence of metastases is associated with low oxygen tension in the primary tumour. The purpose of the study reported here was to search for correlations between incidence of metastases and oxygen tension or vascular density in the same population of patients. Thirty-eight consecutive patients with squamous cell carcinoma of the uterine cervix were included in a prospective study. Pelvic, iliac and retroperitoneal lymph node metastases were detected by magnetic resonance imaging at the time of initial diagnosis. Oxygen tension was measured polarographically using the Eppendorf pO2 Histograph 6650. Vascular density was determined by histological examination of tumour biopsies. The primary tumours of the patients with metastases (n = 19) were more poorly oxygenated than those of the patients without metastases (n = 19). Thus, the fractions of the pO2 readings resulting in values below 5 mmHg and 10 mmHg were significantly higher for the former group of patients than for the latter (P = 0.03 and 0.02 respectively). In contrast, the vascular density of the primary tumour was not significantly different for the two groups of patients. The present study suggests that a high incidence of metastases in squamous cell carcinoma of the uterine cervix is associated with poor oxygenation of the primary tumour and not with a high vascular density.


British Journal of Cancer (1998) 78(6), 822–827
© 1998 Cancer Research Campaign

### Tumour hypoxia and vascular density as predictors of metastasis in squamous cell carcinoma of the uterine cervix

**K Sundfør[1], H Lyng[2] and EK Rofstad[2]**

Departments of [1]Gynaecology and [2]Biophysics, The Norwegian Radium Hospital, Montebello, N-0310 Oslo, Norway

**Summary** Some clinical studies involving several histological types of cancer have suggested that high vascular density in the primary tumour promotes metastasis. Other studies have suggested that a high incidence of metastases is associated with low oxygen tension in the primary tumour. The purpose of the study reported here was to search for correlations between incidence of metastases and oxygen tension or vascular density in the same population of patients. Thirty-eight consecutive patients with squamous cell carcinoma of the uterine cervix were included in a prospective study. Pelvic, iliac and retroperitoneal lymph node metastases were detected by magnetic resonance imaging at the time of initial diagnosis. Oxygen tension was measured polarographically using the Eppendorf $pO_2$ Histograph 6650. Vascular density was determined by histological examination of tumour biopsies. The primary tumours of the patients with metastases ($n$ = 19) were more poorly oxygenated than those of the patients without metastases ($n$ = 19). Thus, the fractions of the $pO_2$ readings resulting in values below 5 mmHg and 10 mmHg were significantly higher for the former group of patients than for the latter ($P$ = 0.03 and 0.02 respectively). In contrast, the vascular density of the primary tumour was not significantly different for the two groups of patients. The present study suggests that a high incidence of metastases in squamous cell carcinoma of the uterine cervix is associated with poor oxygenation of the primary tumour and not with a high vascular density.

**Keywords:** cervix carcinoma; metastasis; oxygen tension; vascular density

The process of metastasis, i.e. the spread of malignant tumour cells from the primary neoplasm to regional or distant sites, is composed of a cascade of linked, sequential and highly selective steps involving multiple host–tumour interactions (Hart et al, 1989; Fidler, 1990). These steps include invasion of tumour cells into blood vessels, survival in the blood circulation, arrest in the capillary bed of a secondary organ, extravasation into the secondary organ interstitium and parenchyma, and tumour cell proliferation and angiogenesis in the secondary organ (Liotta and Stetler-Stevenson, 1991; Nicolson, 1993; Fidler and Ellis, 1994). Each step in the process is rate-limiting; failure to complete any one step prevents tumour cells from producing metastases (Ellis and Fidler, 1996). Biological properties of primary tumours correlating with the probability of metastasis may be hard to identify owing to the complexity of the metastatic process. However, prognostic indicators predicting tumour metastasis are highly needed, as most deaths from cancer result from regional or distant metastases (Liotta, 1992).

In the early 1990s, a positive correlation between lymph node metastasis and the vascular density of the primary tumour was demonstrated in invasive breast carcinoma (Weidner et al, 1991). Since then, similar correlations have been observed in a variety of histological types of human cancer (Weidner, 1993, 1995), leading to the suggestion that the rate of angiogenesis may be an independent prognostic indicator in malignant diseases (Vermeulen et al, 1996). In accordance with this suggestion, recent studies of squamous cell carcinoma of the uterine cervix have shown that high vascular density in the primary tumour is correlated with vascular space invasion, lymphatic involvement and pelvic lymph node metastasis (Wiggins et al, 1995; Bremer et al, 1996; Hawighorst et al, 1997), and may predict low recurrence-free and overall survival probabilities (Schlenger et al, 1995). The metastatic propensity of a tumour may be influenced by the angiogenic potential of the tumour cells by two independent mechanisms: high vascular density in the primary tumour may enhance the opportunity of tumour cells to gain access to the blood circulation; and elevated capacity to induce neovascularization may increase the probability of tumour cells trapped in secondary organ capillary beds to give rise to macroscopic tumour growth (Blood and Zetter, 1990; Mahadevan and Hart, 1990; Fidler and Ellis, 1994).

The development of metastases in human cancer has also been shown to be correlated with the oxygenation status of the primary tumour. Studies of soft-tissue sarcoma have shown that low oxygen tension in the primary tumour is associated with a high incidence of pulmonary metastases (Brizel et al, 1996). Tumour hypoxia has been shown to promote lymph–vascular space involvement and parametrial infiltration in squamous cell carcinoma of the uterine cervix (Höckel et al, 1996). Moreover, positive correlations between the lactate concentration of the primary tumour and the incidence of lymph node metastases have been demonstrated in cervical carcinoma (Schwickert et al, 1995) and in carcinoma of the head and neck (Walenta et al, 1997). High lactate

*Received 8 January 1998*
*Revised 4 March 1998*
*Accepted 17 March 1998*

*Correspondence to:* EK Rofstad, Department of Biophysics, Institute for Cancer Research, The Norwegian Radium Hospital, Montebello, N-0310 Oslo, Norway concentration is indicative of extensive anaerobic metabolism and hence poor oxygenation in tumour tissue (Vaupel et al, 1989). Tumour hypoxia can cause increased expression of several selected genes, including the genes encoding angiogenesis and metastasis-promoting proteins, and hence enhance the metastatic propensity of tumours (Brown and Giaccia, 1994; Dachs and Stratford, 1996; Sutherland et al, 1996; Adams et al, 1997).

The investigations showing correlations between low oxygen tension in the primary tumour and high incidence of metastases are apparently inconsistent with those showing correlations between high vascular density in the primary tumour and high incidence of metastases, as low oxygen tension in tumours is expected to be a result of poor oxygen supply owing to inadequate vascularization (Vaupel et al, 1989; Gulledge and Dewhirst, 1996). Studies comparing the oxygenation and the vascularization of the primary tumour with the incidence of metastases in the same group of patients have not been published so far for any histological type of cancer. Measurements of oxygen tension and vascular density in the primary tumours of patients with squamous cell carcinoma of the uterine cervix are reported in the present communication. The purpose of the work was to search for correlations between the incidence of metastases on the one hand and oxygen tension and vascular density on the other.

**Figure 1** Frequency distributions of $pO_2$ of a poorly oxygenated (**A**) and a well-oxygenated (**B**) primary tumour of patients with squamous cell carcinoma of the uterine cervix. The two $pO_2$ frequency distributions are significantly different ($P < 0.001$)

### MATERIALS AND METHODS

### Patients

Thirty-eight consecutive patients with squamous cell carcinoma of the uterine cervix were included in the study. The inclusion criteria required that (a) the largest diameter of the primary tumour, determined from pretreatment magnetic resonance images, was 2 cm or more, (b) the patients were less than 70 years of age and (c) the patients met the criteria for anaesthesia function class ASA I or ASA II. Clinical stage was determined according to the FIGO criteria. The numbers of patients in the different stages were seven (Ib), one (IIa), 21 (IIb), seven (IIIb) and two (IVa).

The metastatic status of the patients was assessed at the time of initial diagnosis. Pelvic, iliac and retroperitoneal lymph node metastases were detected by magnetic resonance imaging. Lymph nodes were considered pathological when the minimal axial diameter was 10 mm or more or when the minimal axial diameter was 8–10 mm and the lymph nodes were round. These criteria have been shown to give high sensitivity, specificity, accuracy and predictive value (Jager et al, 1996). Distant metastases were localized by radiographic examination, computerized tomography imaging or magnetic resonance imaging and confirmed by biopsy or punction cytology. Informed consent was obtained from all patients. The study was approved by the local ethical committee.

### Oxygen tension

Tumour oxygen tension ($pO_2$) was measured before treatment using a polarographic needle electrode (Eppendorf $pO_2$ Histograph 6650) (Sundfør et al, 1997). The electrode was moved automatically through the tissue in preset steps of 0.7 or 1.0 mm. Each forward step was followed by a backward step of 0.3 mm, leading to a distance of 0.4 or 0.7 mm between each $pO_2$ reading. A total of 57–252 readings in 2–6 tracks were performed in each tumour. The tracks were located peripherally (clock positions 3, 6, 9 and 12) or centrally and were directed perpendicularly to the tumour surface. The track lengths were determined by the tumour size, measured by analysing magnetic resonance images. A $pO_2$ frequency distribution was generated for each tumour by pooling the data from the individual tracks. Heart rate, arterial blood pressure and arterial $HbO_2$ saturation were recorded throughout the $pO_2$ measurements.

### Vascular density

A needle biopsy, 1 × 18 mm in size, was taken from each electrode track, leading to 2–6 biopsies per tumour. The biopsies were fixed in phosphate-buffered 4% paraformaldehyde, embedded in paraffin casts and cut in the length direction to 5-μm-thick sections. The sections were stained with haematoxylin and eosin and subjected to analysis using a projecting light microscope (Lyng et al, 1991). Blood vessels were identified as a lumen encircled by either a thick vessel wall or a lining of endothelial cells, using a magnification of × 410. Vascular density, i.e. the number of vessel profiles per $mm^2$ of tumour tissue, was recorded for each biopsy. Mean tumour vascular density was defined as the mean of the values pertaining to the individual biopsies. The three regions of 0.5 $mm^2$ having the highest vascular density were selected for each tumour and analysed separately. The maximum vascular density of a tumour was defined as the mean of the values pertaining to these regions.

© Cancer Research Campaign 1998

**Figure 2** The fraction of $pO_2$ readings giving values below 5 mmHg (**A**) and 10 mmHg (**B**) in the primary tumour of patients with squamous cell carcinoma of

### Statistical analysis

The Mann–Whitney rank sum test was used to compare two $pO_2$ frequency distributions and to investigate whether $pO_2$ and vascular parameters differed between metastatic and non-metastatic tumours. Correlation between maximum vascular density and mean vascular density was searched for by liner regression analysis. A significance criterion of $P < 0.05$ was used in all analyses.

### RESULTS

The $pO_2$ frequency distributions differed substantially among tumours in different patients, although the tumours were highly heterogeneous in $pO_2$. This is illustrated in Figure 1, which shows the $pO_2$ frequency distributions of a poorly oxygenated and a well-oxygenated tumour. The patient with the poorly oxygenated tumour had developed regional metastases when the $pO_2$ measurements were performed, whereas the patient with the well-oxygenated tumour had not. Tumour oxygenation status did not correlate with clinical stage or histological grade. Significant differences in oxygenation status between small and large tumours, between endophytic and exophytic tumours, between tumours in patients with low and high haemoglobin concentrations or between tumours in premenopausal and post-menopausal women were not found.

Nineteen of the 38 patients had developed regional metastases at the time of initial diagnosis. Three of these patients also showed distant metastases, whereas metastases could not be detected in the remaining 19 patients. The primary tumours of the patients with metastases were more poorly oxygenated than those of the patients without metastases. The difference between the $pO_2$ frequency distributions of the two groups of patients was most pronounced at low $pO_2$ values, i.e. $pO_2$ values compatible with hypoxia-induced radiation resistance and hypoxia-induced gene expression. The fractions of the $pO_2$ readings resulting in values below 5 mmHg and 10 mmHg for the patients with metastases and the patients without metastases are compared in Figure 2. The patients with metastases showed significantly higher fractions than those without metastases, whether the cut-off value was 5 mmHg ($P = 0.03$) or 10 mmHg ($P = 0.02$). In contrast, median $pO_2$ was not significantly different for the patients with metastases and the patients without metastases ($P = 0.38$).

The vascular density differed substantially among the 38 primary tumours. The mean and the maximum values (number per $mm^2$) ranged from 7 to 37 and from 11 to 71 respectively. Figure 3 shows a plot of maximum vascular density vs mean vascular density. There was a strong correlation between the two vascular parameters ($r^2 = 0.62$; $P < 0.001$), i.e. the tumours which showed a high maximum vascular density also showed a high mean vascular density and vice versa. Figure 4 compares the patients with metastases and the patients without metastases with respect to mean and maximum tumour vascular density. The patients with metastases tended to show lower mean values than those without metastases. However, the difference was not statistically significant ($P = 0.10$). Maximum vascular density was similar for the patients with metastases and the patients without metastases ($P = 0.45$).

The patients with metastases showed lower tumour oxygen tensions (Figure 2) and tended to show lower mean tumour vascular densities (Figure 4) than those without metastases. However, the oxygenation of the tumours was not strongly related to the vascularity; statistically significant correlations were not found when the fraction of $pO_2$ readings below 5 mmHg, the fraction of $pO_2$ readings below 10 mmHg or median $pO_2$ was plotted vs mean or maximum vascular density.

### DISCUSSION

Recent studies involving several histogical types of human cancer, including squamous cell carcinoma of the uterine cervix, have suggested that high vascular density in the primary tumour is associated with a high incidence of metastases (Weidner, 1995; Wiggins et al, 1995; Bremer et al, 1996; Vermeulen et al, 1996; Hawighorst et al, 1997). Other studies, apparently inconsistent

 © Cancer Research Campaign 1998

**Figure 3** Maximum vascular density vs mean vascular density in the primary tumour of patients with squamous cell carcinoma of the uterine cervix. The curve was fitted to the data by linear regression analysis ($r^2 = 0.62$; $P < 0.001$)

with those mentioned above, have suggested that tumour hypoxia may promote metastasis in cervical carcinoma (Schwickert et al 1995; Höckel et al, 1996), as well as in soft-tissue sarcoma (Brizel et al, 1996) and carcinoma of the head and neck (Walenta et al, 1997). A prospective study comparing the potential of low oxygen tension and high vascular density in predicting metastasis in cervical carcinoma is reported here. The study was restricted to patients having primary tumours with a largest diameter of at least 2 cm to enable reliable $pO_2$ measurements. The tumours of the patients with metastases showed lower oxygen tensions and tended to show lower vascular densities than those of the patients without metastases. Consequently, the present study suggests that a high incidence of metastases in squamous cell carcinoma of the uterine cervix is associated with poor oxygenation of the primary tumour and not with a high vascular density.

Our study is at variance with the many clinical studies that have demonstrated positive correlations between the vascular density of the primary tumour and the incidence of regional or distant metastases (Weidner, 1993, 1995). In many of the studies, specific endothelial stains were used to highlight the vessels before the vascular density was scored at low magnifications (× 100–200). Preliminary studies performed in our laboratory have shown that many tumour vessels are inadequately stained by using antibodies against factor VIII or CD31 antigens. Similar observations have been reported by others studying the vascularization of squamous cell carcinoma of the uterine cervix (Kainz et al, 1995; Wiggins et al, 1995; Dinh et al, 1996). The problem of varied staining was avoided in the present work by assessing the vascular density at a high magnification (× 410) without the use of specific endothelial stains. The main disadvantage of our method is that the vessel counting is time-consuming. Repeated analyses of the same sections have shown that the reproducibility of the method used here is at least as good as that of methods based on the use of specific endothelial stains. Consequently, the discrepancy between our study and the studies that have demonstrated positive correlations between the vascular density of the primary tumour and the incidence of regional or distant metastases can probably not be attributed to different methods of vessel identification.

In many of the studies that have demonstrated positive correlations between vascular density and incidence of metastases, the vascular density was scored in vascular hotspots of the primary tumour, i.e. in selected regions with elevated vascular density (Weidner et al, 1991; Vermeulen et al, 1996). The discrepancy between our study and these studies cannot be attributed to the use of different procedures for assessment of vascular density either. The parameter termed maximum vascular density in our study is probably closely related to the hotspot vascular density, and the maximum vascular density was similar for the patients with metastases and the patients without metastases. Moreover, our study

**Figure 4** Mean vascular density (**A**) and maximum vascular density (**B**) in the primary tumour of patients with squamous cell carcinoma of the uterine cervix. The columns and bars represent means ± s.e. of 19 patients with metastases (Met +) and 19 patients without metastases (Met –)

revealed a strong correlation between maximum vascular density and mean vascular density, and the mean vascular density tended to be lower for the patients with metastases than for those without metastases.

It should also be noticed that our study is not the only one that has failed to demonstrate a positive correlation between metastasis and vascular density. The initial observation of Weidner et al (1991) that high vascular density in the primary tumour may promote metastasis in breast carcinoma was not confirmed in two recent, extensive studies. Axelsson et al (1995) analysed tumour specimens from 220 patients with breast carcinoma and found no correlation between the vascular density of the primary tumour and metastasis-free survival or overall survival. Similarly, Goulding et al (1995) examined specimens from the primary tumour of 165 breast cancer patients and found no correlation between tumour vascularity and incidence of distant metastases or overall survival probability. Similar observations have also been reported for other histological types of cancer, including colorectal carcinoma (Bossi et al, 1995), malignant melanoma (Carnochan et al, 1991; Busam et al, 1995) and squamous cell carcinoma of the head and neck (Leedy et al, 1994).

The main conclusion of our study is that tumour hypoxia may promote lymph node metastasis in squamous cell carcinoma of the uterine cervix. This conclusion is consistent with the observations that low oxygen tension and high lactate concentration are associated with a high incidence of metastases in human cancer (Schwickert et al, 1995; Brizel et al, 1996; Höckel et al, 1996; Walenta et al, 1997). Studies of experimental tumours have also shown that hypoxia may promote the development of metastatic disease. Thus, exposure of tumour cells to hypoxia in vitro before intravenous inoculation in mice has been shown to increase the frequency of lung colonies in murine tumours (Young et al, 1988) and human melanoma xenografts (Rofstad and Danielsen, 1998).

Tumour stage, grade and volume as well as patient age and blood counts are important prognostic factors in squamous cell carcinoma of the uterine cervix (Kapp et al, 1983). Correlations between tumour oxygenation status and clinical stage, histological grade, tumour volume, patient age or haemoglobin concentration in peripheral blood were not found in the present study, consistent with previous reports (Höckel et al, 1991, 1996). These observations suggest that tumour oxygenation status is an independent prognostic indicator predicting lymph node metastasis in cervix carcinoma.

Several mechanisms can cause a correlation between tumour hypoxia and metastasis in cervical carcinoma. Studies of oncogenically transformed rodent fibroblasts have shown that hypoxia can select for cell subpopulations that are deficient in the apoptotic programme owing to mutations in the *p53* tumour-suppressor gene (Graeber et al, 1996). It has also been shown that the expression of viral oncoproteins in human cervical epithelial cells can increase their sensitivity to hypoxia-induced apoptosis and that long-term culture under hypoxic conditions can select for cell variants that have lost their apoptotic potential (Kim et al, 1997). These observations, together with the observation that cell lines derived from human papillomavirus-associated human cervical carcinomas show reduced sensitivity to hypoxia-induced apoptosis, have led to the suggestion that hypoxia provides a physiological pressure in cervical carcinoma for the expansion of cell subpopulations with a survival advantage to adverse conditions and, hence, with an increased metastatic potential (Kim et al, 1997).

Evidence is accumulating that hypoxia may also promote the malignant progression and metastasis of tumours through effects on signal transduction pathways and by regulating the transcription of various genes (Sutherland et al, 1996; Adams et al, 1997). Several specific genes show altered expression at oxygen tensions below 10 mmHg, and some of the genes encode proteins involved in the metastatic process (Dachs and Stratford, 1996; Sutherland et al, 1996). These proteins include cell adhesion molecules, protein-degrading enzymes and positive and negative angiogenesis factors (Brown and Giaccia, 1994; Dachs and Stratford, 1996). As an example, the expression of vascular endothelial growth factor, a positive angiogenesis factor known to be involved in the angiogenesis of cervical carcinoma (Guidi et al, 1995) is up-regulated significantly under hypoxic conditions both in vitro and in vivo (Plate et al, 1992; Shweiki et al, 1992; Waleh et al, 1995; Mazure et al, 1996).

In conclusion, the data presented here suggest that lymph node metastasis in squamous cell carcinoma of the uterine cervix is associated with poor oxygenation of the primary tumour and not with extensive vascularization. Tumour hypoxia may, therefore, be a useful indicator of aggressive disease, and $p\mathrm{O}_2$ measurements may help to select those patients who have the highest probabilities of developing regional and distant metastases.

### ACKNOWLEDGEMENTS

Financial support was received from The Bothner Foundation for Cancer Research and The Norwegian Cancer Society.

### REFERENCES

Adams GE, Hasan NM and Joiner MC (1997) Radiation, hypoxia and genetic stimulation: implications for future therapies. *Radiother Oncol* **44**: 101–109

Axelsson K, Ljung BME, Moore DH, Thor AD, Chew KL, Edgerton SM, Smith HS and Mayall BH (1995) Tumor angiogenesis as a prognostic assay for invasive ductal breast carcinoma. *J Natl Cancer Inst* **87**: 997–1008

Blood CH and Zetter BR (1990) Tumor interactions with the vasculature: angiogenesis and tumor metastasis. *Biochim Biophys Acta* **1032**: 89–118

Bossi P, Viale G, Lee AKC, Alfano RM, Coggi G and Bosari S (1995) Angiogenesis in colorectal tumors: microvessel quantitation in adenomas and carcinomas with clinicopathological correlations. *Cancer Res* **55**: 5049–5053

Bremer GL, Tiebosch ATMG, van der Putten HWHM, Schouten HJA, de Haan J and Arends J-W (1996) Tumor angiogenesis: an independent prognostic parameter in cervical cancer. *Am J Obstet Gynecol* **174**: 126–131

Brizel DM, Scully SP, Harrelson JM, Layfield LJ, Bean JM, Prosnitz LR and Dewhirst MW (1996) Tumor oxygenation predicts for the likelihood of distant metastases in human soft tissue sarcoma. *Cancer Res* **56**: 941–943

Brown JM and Giaccia AJ (1994) Tumour hypoxia: the picture has changed in the 1990s. *Int J Radiat Biol* **65**: 95–102

Busam KJ, Berwick M, Blessing K, Fandrey K, Kang S, Karaoli T, Fine J, Cochran AJ, White WL, Rivers J, Elder DE, Wen DRP, Heyman BH and Barnhill RL (1995) Tumor vascularity is not a prognostic factor for malignant melanoma of the skin. *Am J Pathol* **147**: 1049–1056

Carnochan P, Briggs JC, Westbury G and Davies AJ (1991) The vascularity of cutaneous melanoma: a quantitative histological study of lesions 0.85–1.25 mm in thickness. *Br J Cancer* **64**: 102–107

Dachs GU and Stratford IJ (1996) The molecular response of mammalian cells to hypoxia and the potential for exploitation in cancer therapy. *Br J Cancer* **74** (suppl. XXVII): s126–s132

Dinh TV, Hannigan EV, Smith ER, Hove MJ, Chopra V and To T (1996) Tumor angiogenesis as a predictor of recurrence in stage Ib squamous cell carcinoma of the cervix. *Obstet Gynecol* **87**: 751–754

Ellis LM and Fidler IJ (1996) Angiogenesis and metastasis. *Eur J Cancer* **32A**: 2451–2460

Fidler IJ (1990) Critical factors in the biology of human cancer metastasis: 28th GHA Clowes memorial award lecture. *Cancer Res* **50**: 6130–6138

Fidler IJ and Ellis LM (1994) The implications of angiogenesis for the biology and therapy of cancer metastasis. *Cell* **79**: 185–188

Goulding H, Nik Abdul Rashid NF, Robertson JF, Bell JA, Elston CW, Blamey RW and Ellis IO (1995) Assessment of angiogenesis in breast carcinoma: an important factor in prognosis? *Human Pathol* **26**: 1196–1200

© Cancer Research Campaign 1998

Graeber TG, Osmanian C, Jacks T, Housman DE, Koch CJ, Lowe SW and Giaccia AJ (1996) Hypoxia-mediated selection of cells with diminished apoptotic potential in solid tumours. *Nature* **379**: 88–91

Guidi AJ, Abu-Jawdeh G, Berse B, Jackman RW, Tognazzi K, Dvorak HF and Brown LF (1995) Vascular permeability factor (vascular endothelial growth factor) expression and angiogenesis in cervical neoplasia. *J Natl Cancer Inst* **87**: 1237–1245

Gulledge CJ and Dewhirst MW (1996) Tumor oxygenation: a matter of supply and demand. *Anticancer Res* **16**: 741–750

Hart IR, Goode NT and Wilson RE (1989) Molecular aspects of the metastatic cascade. *Biochim Biophys Acta* **989**: 65–84

Hawighorst H, Knapstein PG, Weikel W, Knopp MV, Zuna I, Knof A, Brix G, Schaeffer U, Wilkens C, Schoenberg SO, Essig M, Vaupel P and van Kaick G (1997) Angiogenesis of uterine cervical carcinoma: characterization by pharmacokinetic magnetic resonance parameters and histological microvessel density with correlation to lymphatic involvement. *Cancer Res* **57**: 4777–4786

Höckel M, Schlenger K, Knoop C and Vaupel P (1991) Oxygenation of carcinomas of the uterine cervix: evaluation by computerized $O_2$ tension measurements. *Cancer Res* **51**: 6098–6102

Höckel M, Schlenger K, Aral B, Mitze M, Schäffer U and Vaupel P (1996) Association between tumor hypoxia and malignant progression in advanced cancer of the uterine cervix. *Cancer Res* **56**: 4509–4515

Jager GJ, Barentsz JO, Oosterhof GO, Witjes JA and Ruijs SJH (1996) Pelvic adenopathy in prostatic and urinary bladder carcinoma: MR imaging with a three-dimensional T1-weighted magnetization-prepared–rapid gradient-echo sequence. *Am J Roentgenol* **167**: 1503–1507

Kainz C, Speiser P, Wanner C, Obermair A, Tempfer C, Sliutz G, Reinthaller A and Breitenecker G (1995) Prognostic value of tumour microvessel density in cancer of the uterine cervix stage IB to IIB. *Anticancer Res* **15**: 1549–1552

Kapp DS, Fischer D, Gutierrez E, Kohorn EI and Schwartz PE (1983) Pretreatment prognostic factors in carcinoma of the uterine cervix: a multivariable analysis of the effect of age, stage, histology and blood counts on survival. *Int J Radiat Oncol Biol Phys* **9**: 445–455

Kim CY, Tsai MH, Osmanian C, Graeber TG, Lee JE, Giffard RG, DiPaolo JA, Peehl DM and Giaccia AJ (1997) Selection of human cervical epithelial cells that possess reduced apoptotic potential to low-oxygen conditions. *Cancer Res* **57**: 4200–4204

Leedy DA, Trune DR, Kronz JD, Weidner N and Cohen JI (1994) Tumor angiogenesis, the p53 antigen, and cervical metastasis in squamous carcinoma. *Otolaryngol Head Neck Surg* **111**: 417–422

Liotta LA (1992) Cancer cell invasion and metastasis. *Sci Am* **266**: 34–41

Liotta LA and Stetler-Stevenson WG (1991) Tumor invasion and metastasis: an imbalance of positive and negative regulation. *Cancer Res* **51**: 5054s–5459s

Lyng H, Monge OR, Bøhler PJ and Rofstad EK (1991) Temperature distribution in locally advanced breast carcinoma during hyperthermic treatment: relationship to perfusion, vascular density, and histology. *Int J Radiat Oncol Biol Phys* **21**: 423–430

Mahadevan V and Hart IR (1990) Metastasis and angiogenesis. *Acta Oncol* **29**: 97–103

Mazure NM, Chen EY, Yeh P, Laderoute KR and Giaccia AJ (1996) Oncogenic transformation and hypoxia synergistically act to modulate vascular endothelial growth factor expression. *Cancer Res* **56**: 3436–3440

Nicolson GL (1993) Cancer progression and growth: relationship of paracrine and autocrine growth mechanisms to organ preference of metastasis. *Exp Cell Res* **204**: 171–180

Plate KH, Breier G, Weich HA and Risau W (1992) Vascular endothelial growth factor is a potential tumour angiogenesis factor in human gliomas in vivo. *Nature* **359**: 845–848

Rofstad EK and Danielsen T (1998) Hypoxia-induced metastasis of human melanoma cells: involvement of vascular endothelial growth factor-mediated angiogenesis. *Cancer Res* (submitted)

Schlenger K, Höckel M, Mitze M, Schaeffer U, Weikel W, Knapstein PG and Lambert A (1995) Tumor vascularity: a novel prognostic factor in advanced cervical carcinoma. *Gynecol Oncol* **59**: 57–66

Schwickert G, Walenta S, Sundfør K, Rofstad EK and Mueller-Klieser W (1995) Correlation of high lactate levels in human cervical cancer with incidence of metastasis. *Cancer Res* **55**: 4757–4759

Shweiki D, Itin A, Soffer D and Keshet E (1992) Vascular endothelial growth factor induced by hypoxia may mediate hypoxia-initiated angiogenesis. *Nature* **359**: 843–845

Sundfør K, Lyng H, Kongsgård U, Tropé C and Rofstad EK (1997) Polarographic measurement of $pO_2$ in cervix carcinoma. *Gynecol Oncol* **64**: 230–236

Sutherland RM, Ausserer WA, Murphy BJ and Laderoute KR (1996) Tumor hypoxia and heterogeneity: challenges and opportunities for the future. *Semin Radiat Oncol* **6**: 59–70

Vaupel P, Kallinowski F and Okunieff P (1989) Blood flow, oxygen and nutrient supply, and metabolic microenvironment of human tumors: a review. *Cancer Res* **49**: 6449–6465

Vermeulen PB, Gasparini G, Fox SB, Toi M, Martin L, McCulloch P, Pezzella F, Viale G, Weidner N, Harris AL and Dirix LY (1996) Quantification of angiogenesis in solid human tumours: an international consensus on the methodology and criteria of evaluation. *Eur J Cancer* **32A**: 2474–2484

Waleh NS, Brody MD, Knapp MA, Mendonca HL, Lord EM, Koch CJ, Laderoute KR and Sutherland RM (1995) Mapping of the vascular endothelial growth factor-producing hypoxic cells in multicellular tumor spheroids using a hypoxia-specific marker. *Cancer Res* **55**: 6222–6226

Walenta S, Salameh A, Lyng H, Evensen JF, Mitze M, Rofstad EK and Mueller-Klieser W (1997) Correlation of high lactate levels in head and neck tumors with incidence of metastasis. *Am J Pathol* **150**: 409–415

Weidner N (1993) Tumor angiogenesis: review of current applications in tumor prognostication. *Semin Diagn Pathol* **10**: 302–313

Weidner N (1995) Intratumor microvessel density as a prognostic factor in cancer. *Am J Pathol* **147**: 9–19

Weidner N, Semple JP, Welch WR and Folkman J (1991) Tumor angiogenesis and metastases – correlation in invasive breast carcinoma. *New Engl J Med* **324**: 1–8

Wiggins DL, Granai CO, Steinhoff MM and Calabresi P (1995) Tumor angiogenesis as a prognostic factor in cervical carcinoma. *Gynecol Oncol* **56**: 353–356

Young SD and Hill RP (1988) Hypoxia induces DNA overreplication and enhances metastatic potential of murine tumor cells. *Proc Natl Acad Sci USA* **85**: 9533–9537

© Cancer Research Campaign 1998